# Alum triggers infiltration of human neutrophils ex vivo and causes lysosomal destabilization and mitochondrial membrane potential‐dependent NET‐formation

**DOI:** 10.1096/fj.202001413R

**Published:** 2020-08-29

**Authors:** Manuel Reithofer, Jasmine Karacs, Johanna Strobl, Claudia Kitzmüller, Dominika Polak, Katharina Seif, Meder Kamalov, Christian F. W. Becker, Georg Greiner, Klaus Schmetterer, Georg Stary, Barbara Bohle, Beatrice Jahn‐Schmid

**Affiliations:** ^1^ Institute of Pathophysiology and Allergy Research Center for Pathophysiology, Infectiology and Immunology Medical University of Vienna Vienna Austria; ^2^ Department of Dermatology Medical University of Vienna Vienna Austria; ^3^ Department of Surgery Medical University of Vienna Vienna Austria; ^4^ Institute of Biological Chemistry Department of Chemistry University of Vienna Vienna Austria; ^5^ Department of Laboratory Medicine Medical University of Vienna Vienna Austria; ^6^ Ludwig Boltzmann Institute for Rare and Undiagnosed Diseases Vienna Austria; ^7^ CeMM Research Center for Molecular Medicine Vienna Austria

**Keywords:** aluminium hydroxide, vaccine, adjuvant, innate response, neutrophils, NET

## Abstract

Aluminium salts have been used in vaccines for decades. However, the mechanisms underlying their adjuvant effect are still unclear. Neutrophils, the first immune cells at the injection site, can release cellular DNA together with granular material, so‐called neutrophil extracellular traps (NETs). In mice, NETs apparently play a role in aluminium hydroxide (alum)‐adjuvant immune response to vaccines. Although no experimental data exist, this effect is assumed to be operative also in humans. As a first step to verify this knowledge in humans, we demonstrate that the injection of alum particles into human skin biopsies ex vivo leads to similar tissue infiltration of neutrophils and NET‐formation. Moreover, we characterized the mechanism leading to alum‐induced NET‐release in human neutrophils as rapid, NADPH oxidase‐independent process involving charge, phagocytosis, phagolysosomal rupture, Ca^2+^‐flux, hyperpolarization of the mitochondrial membrane, and mitochondrial ROS. Extracellular flow and inhibition experiments suggested that no additional energy from oxidative phosphorylation or glycolysis is required for NET‐release. This study suggests a so far unappreciated role for neutrophils in the initial phase of immune responses to alum‐containing vaccines in humans and provides novel insights into bioenergetic requirements of NET‐formation.

**Abbreviations**
2‐DG: 2‐D‐glucose
Alum: aluminium hydroxide
CitH3: citrullinated histone 3
cROS: cellular ROS (NOX‐2 dependent)
DAMP: danger‐associated molecular pattern
ECAR: extracellular acidification rate
LL‐37: cathelicidin peptide 37
MLKL: mixed lineage kinase domain‐like protein
MPO: myeloid peroxidase
mROS: mitochondrial ROS
NALP3: nucleotide oligomerization domain‐like receptor pyrin‐domain‐containing 3
NE: neutrophil elastase
NET: neutrophil extracellular traps
NOX‐2: NADPH‐dependent oxidase 2
OCR: oxygen consumption rate
PAD4: protein arginine deiminase 4
PMA: phorbol‐12‐myristate‐13‐acetate
RIPK 1/3: kinase activity receptor‐interacting protein 1/ 3
ΔΨ_m_: mitochondrial membrane potential

## 1 INTRODUCTION

For almost 90 years aluminium‐based adjuvants have been licensed for vaccines in humans. Aluminum hydroxide, often referred to as alum, still represents the most widely used adjuvant for poorly immunogenic antigens.^1^ Aluminum salts form nanocrystals^2^ and in aqueous environments colloid microparticles^3^ with an extremely high protein‐binding capacity mostly based on electrostatic forces of the strong positive charge of aluminium. Adjuvants in general augment adaptive immune responses by activating innate antigen presenting cells, but the mechanisms underlying the adjuvanticity of aluminum‐based adjuvants are still not entirely clear. Initially, a depot‐effect was claimed, however, antigens are released rapidly^4^ and immune responses remained unchanged when alum was removed within 2 hours after injection.^5^ As sterile aluminium‐containing vaccines lack microbial pathogen associated molecular patterns, induction of endogenous mediators representing damage‐associated molecular patterns (DAMPs) by tissue damage seems likely.^6^, ^7^ For instance, in mice, alum‐induced uric acid has been shown to activate inflammatory monocytes to differentiate into inflammatory dendritic cells^8^ involving the cytosolic nucleotide oligomerization domain (NOD)‐like receptor pyrin‐domain‐containing 3 (NLRP3). However, in vivo studies using different NLRP3‐deficient mice provided controversial results regarding the role of NLRP3.^9^, ^10^, ^11^, ^12^, ^13^, ^14^

In mice, injection of alum initiates a pro‐inflammatory effect within 24 hours. First, neutrophils are recruited. Later, monocytes, macrophages, myeloid, and plasmacytoid dendritic cells appear and trigger specific immune responses by antigen presentation.^13^, ^15^ Two ‐ four hours after injection, alum causes the development of nodules containing fibrin and DNA with features of extracellular traps.^16^ Neutrophils, macrophages, and eosinophils found in close vicinity to such nodules represent possible sources of this extracellular DNA.

Neutrophils have mainly been perceived as first line defense due to their capacity to phagocytose, kill, and degrade pathogens. More recently, their ability to release extracellular traps (NETs) was recognized as an alternative mode to sequester and destroy microbes or to combat pathogens too large to be phagocytosed as fungal hyphae.^17^, ^18^ NETs have been described as filamentous, web‐like decondensed chromatin and/or mitochondrial DNA associated with granular proteins, for example, myeloperoxidase (MPO), neutrophil elastase (NE), or the antimicrobial cationic cathelicidin peptide LL‐37.^17^, ^18^, ^19^, ^20^ The most studied trigger of NET‐formation is phorbol‐12‐myristate‐13‐acetate (PMA). It induces the membrane‐based multiprotein enzyme complex NADPH‐oxidase (NOX2) to produce reactive oxygen species (ROS) and activates the Raf/MEK/ERK and p38 MAPK pathways.^21^ PMA‐triggered NET‐induction involves MPO and NE activity to decondensed chromatin.^19^ Disintegration of the nuclear and granular membranes eventually leads to the rupture of the plasma membrane, release of the cell content, and concomitant neutrophil death. This regulated death program differs from apoptosis and has been termed NETosis.^20^ In addition, a NOX2‐independent NET‐release involving mitochondrial ROS production has been described in response to calcium ionophores like ionomycin,^22^ microbes like *Staphylococcus aureus* or *Candida albicans* hyphae,^23^ or microparticles like urate crystals.^24^ The process induced by these stimuli occurs much more rapidly than PMA‐induced NET‐formation and histone hypercitrullination by protein arginine deiminase 4 (PAD4) is thought to be involved in chromatin decondensation.^25^ Although still controversial, activation of the necroptosis signaling pathway with the kinase activity receptor‐interacting proteins 1 and 3 (RIPK1 and RIPK3) and mixed lineage kinase domain‐like protein (MLKL) has been suggested to result in membrane rupture and NET‐release.^26^, ^27^

Recently, intravital imaging of the alum injection site in mice revealed the appearance of neutrophil swarms and extracellular DNA strands as earliest events.^13^ Notably, the adjuvant effect of alum was significantly reduced in PAD4‐deficient mice, which were unable to form NETs. In humans, neutrophils comprise 40%‐60% of peripheral white blood cells as opposed to 10%‐15% in mice, and thus may play an even more important role in the initial steps of alum‐induced immune responses. The capacity of alum to trigger NETs in human neutrophils in vitro has been suggested,^28^ however the underlying mechanisms have not been explored so far. Using a novel human ex vivo model, we demonstrate that injection of alum into skin biopsies leads to infiltration of neutrophils and NET‐formation. We reveal cellular mechanisms underlying alum‐induced NET‐induction and provide novel insights in its bioenergetic requirements.

## 2 MATERIALS AND METHODS

### 2.1 Human skin biopsies and injection of alum

The application of healthy human skin samples from skin reduction operations for ex vivo experiments had been approved by the ethics committee of the Medical University of Vienna (EK 1281/2018). Skin punch biopsies (6 mm in diameter) were performed on skin tissue discarded during abdominoplasty. A total of 40 µL PBS or 40 µL sterile, pyrogen‐free Alu‐Gel‐S correlating to 100 µg Al_2_O_3_ (SERVA, Heidelberg, Germany) were injected intradermally with a 1 mL syringe using a 27G needle. The biopsies were incubated in 6‐well plates with RPMI (Gibco, Invitrogen GmbH, Lofer, Austria) medium for 3 hours at 37°C and 5% CO_2_. Then, the tissue was embedded in Tissue‐Tek optimal cutting temperature (OCT) compound (Sakura Finetek Europe BV, NL) and deep‐frozen in liquid nitrogen. The frozen specimens were stored at −20°C until further processing.

### 2.2 Immunofluorescence staining of skin sections and imaging

For immunofluorescence staining, the frozen OCT‐embedded tissue was cut into 5‐µm sections and mounted on capillary gap microscope slides (Dako, Glostrup, Denmark). After 20 minutes of air‐drying, the cryostat sections were fixed in ice‐cold acetone (Sigma‐Aldrich, St. Louis, Mo., USA) for 10 minutes and stored at –20°C. For staining, the slides were washed three times for 5 minutes with PBS and then, incubated for 30 minutes with human AB‐serum (20% in PBS) to diminish background staining. The primary monoclonal antibodies anti‐citrullinated histone H3 (ab 5103, Abcam, Cambridge, UK), rabbit anti‐neutrophil elastase (ab 131260, Abcam, Cambridge, UK), and mouse anti‐LL‐37 (sc‐166770, Santa Cruz Biotechnology, Dallas, Texas) diluted in 2% bovine serum albumin (BSA; Sigma‐Aldrich, St. Louis, Mo., USA) in PBS (Gibco, Invitrogen GmbH, Lofer, Austria) were applied overnight in a humid chamber at 4°C. Then, slides were washed three times for 5 minutes with PBS and secondary antibodies (anti‐rabbit Alexa 488, anti‐mouse Alexa 568 or anti‐rabbit‐Alexa 647 (all Jackson Immuno Research Inc, West Grove, PA, USA), mouse anti‐CD16 PE (Biolegend, San Diego, CA) were added in 2% BSA/PBS for 45 minutes. Slides were again washed three times for 5 minutes in PBS and DNA staining was performed with 4′,6‐diamidino‐2‐phenylindole (DAPI) for 5 minutes. After a washing step for 5 minutes in PBS, coverslips were mounted onto the slides with fluorescence mounting medium (Dako). Slides were scanned using the digital TissueFAXS imaging system with TissueFAXS software (TissueGnostics GmbH, Vienna, Austria) with 20‐fold magnification. All images were acquired and analyzed using identical hardware and software settings.

### 2.3 Blood donors

The peripheral blood cells used in this study were isolated from healthy individuals. This study had been approved by the ethics committee of the Medical University of Vienna (EK 1488/2017) and all subjects gave written informed consent.

### 2.4 Neutrophil isolation

Neutrophils were isolated from heparinized peripheral blood by Ficoll‐Hypaque gradients (Seromed‐Fakola AG, Basel, Switzerland), dextran sedimentation, and osmotic lysis of remaining erythrocytes as previously described.^29^ Cells were >99% viable as assessed by trypan blue exclusion, and consisted of 92.4% ± 5.1 (mean ± SD; n = 14) CD16^+^CD66b^+^ neutrophils as evaluated by flow cytometry.

### 2.5 Flow cytometric analysis of neutrophils

After saturation of unspecific binding with 20% AB‐serum in PBS containing 0.1% BSA and 0.1% NaN_3_ for 20 minutes at 4°C, neutrophils were stained for 30 minutes at 4°C with fluorescence‐labeled antibodies: CD11b‐APC, CD66b‐PerCP (both eBioscience, San Diego, CA), CD16‐Brilliant Violet V510, and CD64‐Brilliant Violet 421 (both Biolegend, San Diego, CA). Stained cells were analyzed by flow cytometry using a FACS Canto II (BD Biosciences) and FlowJo software (Treestar Inc). For cell counting experiments 20 000 123count eBeads counting beads (Invitrogen, Thermo Fisher Scientific, Waltham, MA) were added prior to analysis and 10 000 beads acquired.

### 2.6 Fluorescence microscopy

Neutrophils were seeded at a density of 1.5 × 10^6^/mL on poly‐D‐lysine (Merck, Sigma‐Aldrich, St. Louis, MO) coated glass coverslips (Ø 12 mm) in 24‐well plates (Costar, Sigma‐Aldrich) and primed with 25 ng/mL GM‐CSF (PeproTech, Rocky Hill, NJ) in RPMI1640 with 2% autologous plasma for 30 minutes at 37°C and 5% CO_2_. The presence of GM‐CSF had no significant influence on the DNA‐release by ionomycin or alum, but seemed to improve the adherence of cells and general cell survival preventing apoptosis and, therefore, was used throughout the study. Then, cells were stimulated with the positive controls 25 nM PMA (Sigma‐Aldrich) and 5 µM ionomycin (Sigma‐Aldrich), or with alum at indicated concentrations for up to 3 hours. Medium alone served as negative control. Cells were fixed in 4% paraformaldehyde in PBS and permeabilized in PBS containing 5 mM NH_4_Cl and 0.2% saponin (Sigma‐Aldrich). The following steps were performed on drops in a humid chamber at RT. Washing of coverslips was performed three times by in PBS for 5 minutes, and blocking with 20% AB‐serum in PBS for 60 minutes. Polyclonal rabbit anti‐myeloperoxidase (MP‐023‐PR6, A.Menarini diagnostics, Vienna, Austria), monoclonal rabbit anti‐neutrophil elastase (ab 131260, Abcam, Cambridge, UK), monoclonal mouse anti‐LL‐37 (sc‐166770, Santa Cruz Biotechnology, Dallas, Texas) and, as secondary antibodies anti‐rabbit Alexa 488 or anti‐mouse Alexa 568 (both Jackson Immuno Research Inc, West Grove, PA, USA) were added for 1 hour. DNA was visualized by incubation of the coverslips at RT with SYTOX orange or DRAQ5 (Invitrogen, Thermo Fisher Scientific) both at 5 µM for 15 minutes. Fluorescence microscopy was performed with an Axioplan 2 microscope (Zeiss) or confocal images taken using an Axiovert 200 microscope (Zeiss) and Z‐stacks analyzed with Volocity Software (Perkin‐Elmer, Waltham, MA).

### 2.7 Quantification of extracellular DNA

A total of 2 × 10^5^ neutrophils in 200 µL were seeded into black flat bottom 96‐well plates (Thermo Fisher) in HBSS medium containing 25 ng/mL GM‐CSF and 5 µM of the cell‐impermeable DNA‐dye SYTOX orange (Thermo Fisher). After 30 minutes of priming, cells were stimulated in triplicates with PMA, ionomycin, alum (Alu‐Gel‐S), or aluminium phosphate (Adju‐Phos; Brenntag, Ballerup, Denmark) at the indicated concentrations. Fluorescence was measured at 575 nm with a TECAN Infinite M1000 fluorescence reader (Tecan, Zürich, CH) every 2 minutes for up to 3 hours. During these experiments the pH of the culture medium remained constant.

### 2.8 Staining of alum with lumogallion or morin

A total of 50 µM lumogallion (Santa Cruz Biotechnology, Dallas, TX) or 250 µM morin (Sigma‐Aldrich) in *aqua bidest* were added to alum and the mixture were incubated overnight in the dark at RT on a lab rotator. Stained alum particles were centrifuged for 10 minutes at 13 000 *g*. The pellets were resuspended in RPMI 1640 containing 2% autologous plasma and immediately used to stimulate neutrophils.

### 2.9 Inhibition experiments

For inhibition experiments, cells were preincubated in the presence of 25 ng/mL GM‐CSF for 30 minutes with the following inhibitors: diphenyleneiodonium (DPI) 20 µM, dinitrophenol (DNP) 750 µM, cytochalasin D 10 µg/mL, oligomycin 10 µM, 2‐deoxy‐D‐glucose 2 mM, EDTA 5 mM, rotenone 10 µM, antimycin 5 µM, ciclosporin 0,5 ng/mL, leupeptin 20 µM, MG‐115 1 µM (all Sigma‐Aldrich, St. Louis, MO, USA), RO‐31‐8220 100 nM, SB203580 10 µM, R406 1 µM, rapamycin 100 nM, wortmannin and chloroquine 10 nM, Nec‐1s 50 µM (all Selleckchem, Houston, TX, USA), bafilomycin A 500 nM, GSK484 1 mM, chymostatin 1 µM, z‐VAD 10 µM, GSK872 100 nM (Cayman Chemicals, Ann Arbor, MI, USA) LDC7559 1 µM (MedChemExpress, NJ, USA), and necrosulfonamid 5 µM (Merck Millipore, Burlington, MA, USA). These concentrations had been optimized in pilot‐experiments. Inhibitors by themselves did not induce DNA‐release from neutrophils (data not shown).

### 2.10 Measurement of intracellular pH

2′,7′‐Bis‐(2‐Carboxyethyl)‐5‐(and‐6)‐Carboxyfluorescein, Acetoxymethyl Ester (BCECF) (Molecular Probes) was used to measure the intracellular pH. Up to 10 × 10^6^ neutrophils in 1 mL PBS with 3 µM BCECF were incubated for 30 minutes at 37°C and 5% CO_2_. Thereafter, cells were harvested and seeded into wells containing HBSS plus 25 ng/mL of GM‐CSF. Cells were incubated for 30 minutes and then, stimulated for 5 minutes with 100 µg/mL alum and fluorescence was assessed immediately by flow cytometry on a BD FACS Canto II and analyzed with FlowJo software.

### 2.11 Isolation of NETs

A total of 6 × 10^6^/1 mL freshly isolated neutrophils were primed with 25 ng/mL GM‐CSF in DMEM for 30 minutes in 24‐well plates (Costar) and then, incubated in duplicates for 3 hours in medium, with 25 nM PMA, 5 µM ionomycin, or alum at a concentration of 100 µg/mL at 37°C and 5% CO_2_. Twenty micro liter of a solution of 500 mU/mL MNase (Sigma‐Aldrich) in distilled water were added to the cultures and incubated for 20 minutes at 37°C. Thereafter, the culture supernatants were harvested and remaining cells were removed by centrifugation at 400 *g* for 5 minutes. These supernatants were collected and termed “NETs.”

### 2.12 Quantification of neutrophil elastase

Elastase activity present in NETs was quantified colorimetrically by conversion of 100 μM peptide substrate N‐(methoxysuccinyl)‐Ala‐Ala‐Pro‐Val 4‐nitroanilide (Sigma‐Aldrich) within 15 minutes at room temperature. Optical density (OD) was measured at 405 nm by a SpectraMax Plus 384 microplate reader (Molecular Devices, Sunnyvale, CA, USA). Results are shown as fold change of OD relative to the OD of medium.

### 2.13 Detection of cytoplasmic and mitochondrial ROS

Chloromethyl‐2′7′‐dichlorodihydrofluorescein diacetate (CM‐H_2_CFDA) (Molecular probes, Life Technologies, Carlsbad, CA, USA) was used to detect the production of cytosolic reactive oxygen species (cROS). A total of 1 × 10^6^ neutrophils in 1 mL were incubated in 24‐well plates for 30 minutes with CM‐H2CFDA in RPMI plus 2% autologous plasma with 25 ng/mL GM‐CSF at 37°C and 5% CO_2._ Cells were harvested, washed with PBS, and stimulated for 30 minutes with the indicated stimuli in HBSS at 37°C and 5% CO_2_. Immediately thereafter, fluorescence was assessed by flow cytometry on a BD FACS Canto II and analyzed with FlowJo software.

MitoSOX Red (Life Technologies) was used to detect the mitochondrial superoxide production in a plate reader assay. A total of 2 × 10^5^ neutrophils in 200 µL were seeded into black flat bottom 96‐well plates (Thermo Fisher) in HBSS medium containing 25 ng/mL GM‐CSF and preincubated for 1 hour with 5 µM MitoSOX Red at 37°C and 5% CO_2_ and then, different stimuli added. Induction of fluorescence by mROS was detected at 2 minutes intervals with a TECAN Infinite M1000 plate reader. The area under the curve was calculated and compared to the medium control.

### 2.14 PCR of mitochondrial and chromosomal genes

NETs were harvested, centrifuged, and supernatants treated for 15 minutes with 60 mU/mL proteinase K (Qiagen, Austin, TX) at 56°C, before DNA was purified with the DNeasy Blood & Tissue kit (Qiagen). A total of 100 ng of DNA were mixed with DNAzyme master mix (Thermo Fisher) and 250 nM primers for the mitochondrial 16S (forward: 5′‐CGCATAAGCCTGCGTCAGATCAA‐3′, reverse: 5′‐TGTGTTGGGTTGACAGTGAGGG‐3′) or the chromosomal 18S gene (forward: 5′‐GTAACCCGTTGAACCCCATT‐3′, reverse: 5′‐CCATCCAATCGGTAGTAGCG‐3′) gene in 20 µL. Additionally, the presence of four mitochondrial genes was tested with following primers: ATP synthase subunit 6 (atp6) (5′‐ATACACAACACTAAAGGACGAACCT‐3′ and 5′‐GAGGCTTACTAGAAGTGTGAAAACG‐3′), cytochrome oxidase c subunit 1 (co1) (5′‐GGAGTCCTAGGCACAGCTCTAA‐3′ and 5′‐GGAGGGTAGAC TGTTCAACCTG‐3′), NADH dehydrogenase subunit 1 (nd1) (5′‐GCATTCCTAATGCTTACCGAAC‐3′ and 5′‐AAGGGTGGAGAGGTTAAAGGAG‐3′), cytochrome oxidase b (cyb) (5′‐CTAGCAGCACTCCACCTCCTAT‐3′ and 5′‐GTTGTCCTCCGATTCAGGTTAG‐3′).^30^ Furthermore, two nuclear genes were also investigated to indicate the presence of nuclear DNA, elongation factor 1 alpha 1 (EF1a1) with the primers: 5′‐CTGAACCATCCAGGCCAAAT‐3′ and 5′‐GCCGTGTGGCAATCCAAT‐3′ and b‐actin with the primers: 5′‐ATCTGGCACCACACCTTCTACAATGAGCTGCG‐3′ and 5′‐CGTCATACTCCTGCTTGCTGATCCACATCTGC‐3′. The DNA was amplified in 40 PCR‐cycles with 98°C for 25 s, 60°C for 1 minutes, 72°C for 45 s and then, analyzed by agarose gel electrophoresis and DNA‐staining with GelRed (Biotium Inc, Fremont, CA, USA) and visualized by Kodak Gel Logic 2200 Imaging System (Kodak, Rochester, NY, USA).

### 2.15 Detection of IL‐8

Released IL‐8 in supernatants of neutrophils after 3 hours of stimulation was determined by the Human IL‐8 ELISA kit (Thermo Fisher).

### 2.16 Immunoblot analysis of citrullinated histone H3

Neutrophils were either stimulated with PMA (25 nM), ionomycin (5 µM), or alum (25 µg/mL) and incubated for 3 hours at 37°C before 500 mU/mL MNase was added and supernatants were collected. Medium was used as negative control. The proteins were separated by reducing SDS‐PAGE and blotted onto Immobilon P membrane (Merck) at 20 V. Unspecific binding was blocked with 5% milk in TBS, before rabbit anti‐histone H3 citrulline R2+R8+R17 (Abcam, 1:1000) was added for 16 hours at 4°C. After washing, HRP‐conjugated anti‐rabbit antibody (1:50 000) was added at RT for 1 hour. After washing, CitH3 was detected with LumigenTMA‐6 (Lumigen Inc, Southfield, MI).

### 2.17 Assessment of lysosomal permeabilzation

Up to 4 × 10^6^ neutrophils in 1 mL PBS were loaded with 100 nM LysoTracker Deep Red for 30 minutes at 37°C and 5% CO_2_. Washed cells were primed for 30 minutes with 25 ng/mL GMCSF in HBSS in 24‐well plates at 1.5 × 10^6^/mL, and stimulated with medium, alum (100 µg/mL), uncharged latex beads (100 µg/mL), positively or negatively charged latex beads (3 µm; 25 µg/mL) and L‐leucyl‐L‐leucine methyl ester (LLOMe; 2.5 µM) as positive control. Fluorescence was assessed immediately by flow cytometry on a BD FACS Canto II and analyzed with FlowJo software.

### 2.18 Bioenergetic assays

To investigate the energy sources involved in NET‐formation, an XF24 Extracellular Flux Analyzer (Seahorse Bioscience, North Billerica, MA, USA) was used to measure the oxygen consumption rate, in most cell types indicating respiration, and the extracellular acidification rate (ECAR) by pH‐changes, indicating glycolysis in the same well. A total of 7.5 × 10^5^ neutrophils were seeded in 630 µL in XF 24‐well cell culture microplates that had been pre‐coated with Cell‐Tak (both Seahorse Bioscience, North Billerica, Massachusetts, USA) and then, primed with 25 ng/mL GM‐CSF in Seahorse XF base medium plus 5 mM glucose (both Seahorse Bioscience) in a CO_2_‐free incubator at 37°C prior to the assay. Samples were analyzed at 37°C. After 45 minutes, 70 µL of alum, ionomycin, PMA, or medium as control were injected. After 2 minutes to mix and homogenize samples and 2 minutes of cell settling, OCR and ECAR were measured every 2 minutes for about 195 minutes.

### 2.19 Determination of mitochondrial membrane potential

A total of 0.5 × 10^6^ neutrophils were primed in 48‐well plates for 30 minutes with GM‐CSF before 10 µg/mL oligomycin (Sigma) were added for 15 minutes. Then, tetramethylrhodamine ethyl ester (TMRE; Abcam; 50 nM), an indicator of the mitochondrial membrane potential ΔΨ_m_ was added. After 20 minutes neutrophils were stimulated with 100 µg/mL alum for 5 minutes and fluorescence immediately analyzed by flow cytometry.

### 2.20 Statistics

In general, the graphs show medians and fold changes, calculated as ratios of stimulated cells to cells in medium control. Means were calculated for data on neutrophil metabolism. For statistical analysis one‐way ANOVA for repeated measurements followed by Dunnett's post tests and paired t tests were performed using GraphPad Prism 5 (GraphPad Software, Inc, La Jolla, CA, USA). Differences were considered statistically significant for values of *P* ≤ .05.

## 3 RESULTS

### 3.1 Ex vivo injection of alum into human skin leads to infiltration of neutrophils and NET‐formation

**Figure 1:**
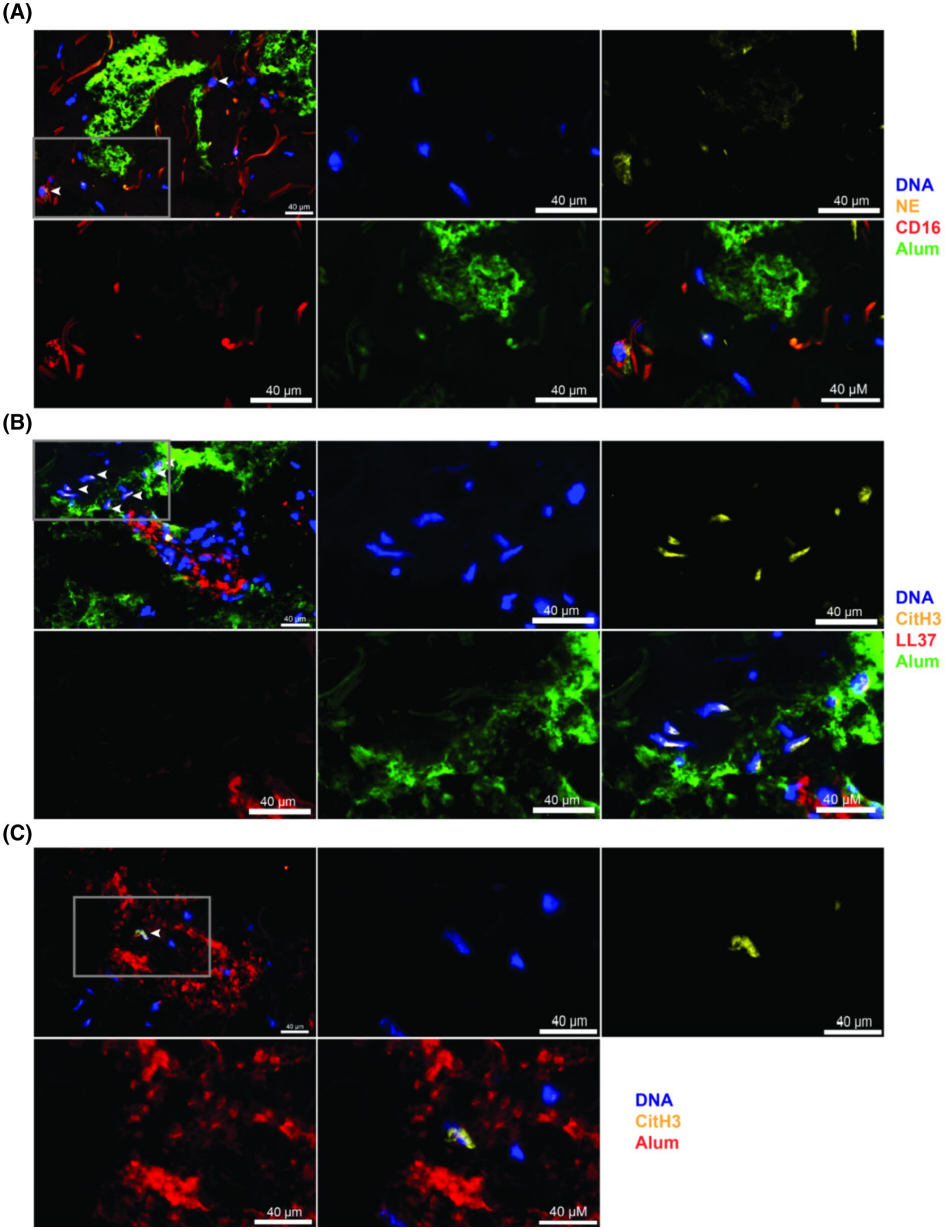
Ex vivo injection of alum into human skin leads to attraction of neutrophils and extracellular trap formation. Fluorescence‐labeled alum (40 µL) was injected intradermally into skin punch biopsies. After 3 h of incubation, skin was flash frozen and sections stained with monoclonal antibodies against NE, LL37, CitH3, CD16, and DAPI nuclear marker. Overviews on the sections shown in (A)‐(C) are presented in the Supporting Figure S1. A, Upper left panel: an area with patches of alum particles (green), and neutrophils (white arrows) identified by CD16 (red) and neutrophil elastase (yellow). The red filamentous structures represent autofluorescent collagen fibers. Detail images of the indicated area (gray frame) are presented in the subsequent panels showing single staining of DNA (blue), NE (yellow), CD16 (red), alum (green), and a merged image. B, Upper left panel: DNA+ (blue) CitH3+ (yellow) extracellular traps (white arrows) in close proximity to alum particles (green) next to a blood vessel (red; staining of LL‐37 in endothelial cells). The subsequent images show the area within the gray frame as close‐up in single and merged colors. C, Upper left panel: an extracellular trap containing DNA (blue) and co‐localized CitH3 (yellow) in close vicinity to alum particles (red). The following images show the framed area in higher magnification. Scale bar 40 µm. One representative experiment out of three is shown

We established a human ex vivo model to investigate the early immune cell response to alum after injection into skin biopsies. Fluorescence‐labeled alum or PBS were injected into biopsies of normal skin and cultured in medium for 3 hours. Using a TissueFAXS imaging system, patches of alum particles were found in the dermis (Figure S1B‐D, Figure 1A‐C). CD16‐ and NE‐ double‐positive neutrophils were the first infiltrating cells (Figure 1A) located outside of vessels and in close vicinity to alum (Figure 1A), indicating infiltration after alum‐induced tissue damage. Importantly, citrullinated histone H3 (CitH3) colocalized with the DNA of infiltrated neutrophils, representing a typical feature of NETs (Figure 1B,C). In contrast, after injection of PBS neutrophils were observed only in the upper dermis, but they were fewer in numbers and without indication of NET‐formation in different three experiments (Figures S1A and S2).

### 3.2 Alum triggers NET‐release in human neutrophils in vitro

**Figure 2:**
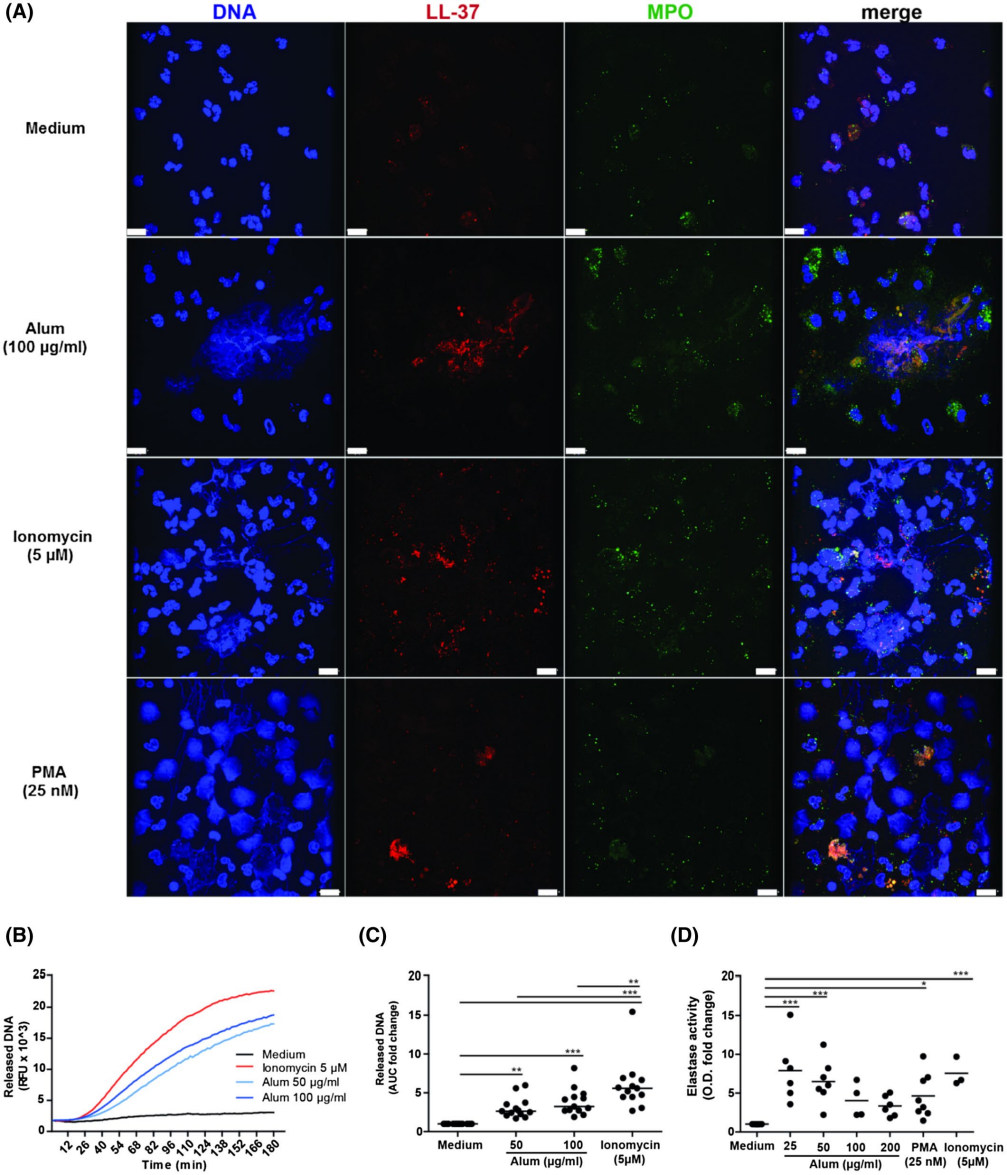
Alum triggers NET‐formation. A, Confocal microscopy of neutrophils after 3 h of incubation with alum, ionomycin, or PMA or medium as negative control. Co‐localization of expelled DNA (blue, left) with the granular components MPO (green, center right) and LL‐37 (red, center left) was observed with all three stimuli. Scale bars, 10 µm. B, The kinetics of alum‐induced release of DNA was measured by fluorescence of SYTOX orange bound to extracellular DNA in plate reader assays. One representative experiment and (C) the accumulated data of 13 independent experiments using neutrophils from different donors (AUC, area under the curve; RFU, relative fluorescence units; horizontal bar, median). D, Elastase activity in the supernatants of MNAse‐treated cultures after 3 h stimulation with the indicated substances was quantified colorimetrically with N‐methoxysuccinyl‐Ala‐Ala‐Pro‐Val‐p‐nitroanilide as substrate and normalized to medium controls (n = 3‐8). *P < .05; **P < .01; ***P < .001 by One‐way ANOVA for repeated measurements followed by Dunnett's post tests

The observation of neutrophil infiltrates and NET‐formation prompted us to perform mechanistic investigations in vitro. Freshly isolated neutrophils were seeded on glass coverslips, primed with GM‐CSF, incubated with medium or stimulated with alum, PMA or ionomycin and evaluated for NET‐formation by fluorescence microscopy (Figure 2A). Medium controls displayed lobular nuclei and the intracellular granular molecules MPO or LL‐37, while NET‐release was observed in both, PMA‐ and ionomycin‐stimulated neutrophils showing disintegrated nuclei and filamentous or web‐like extracellular DNA co‐localizing with MPO or LL‐37. Alum also triggered the release of DNA forming more cloud‐like structures with intense co‐staining of granular components. Interestingly, not all the neutrophils were activated on glass coverslips, but, applying the same conditions, we observed that nearly all neutrophils formed NETs on plastic surfaces of tissue culture plates.

### 3.3 Alum and ionomycin induce NET‐release with similar kinetics

**Figure 3:**
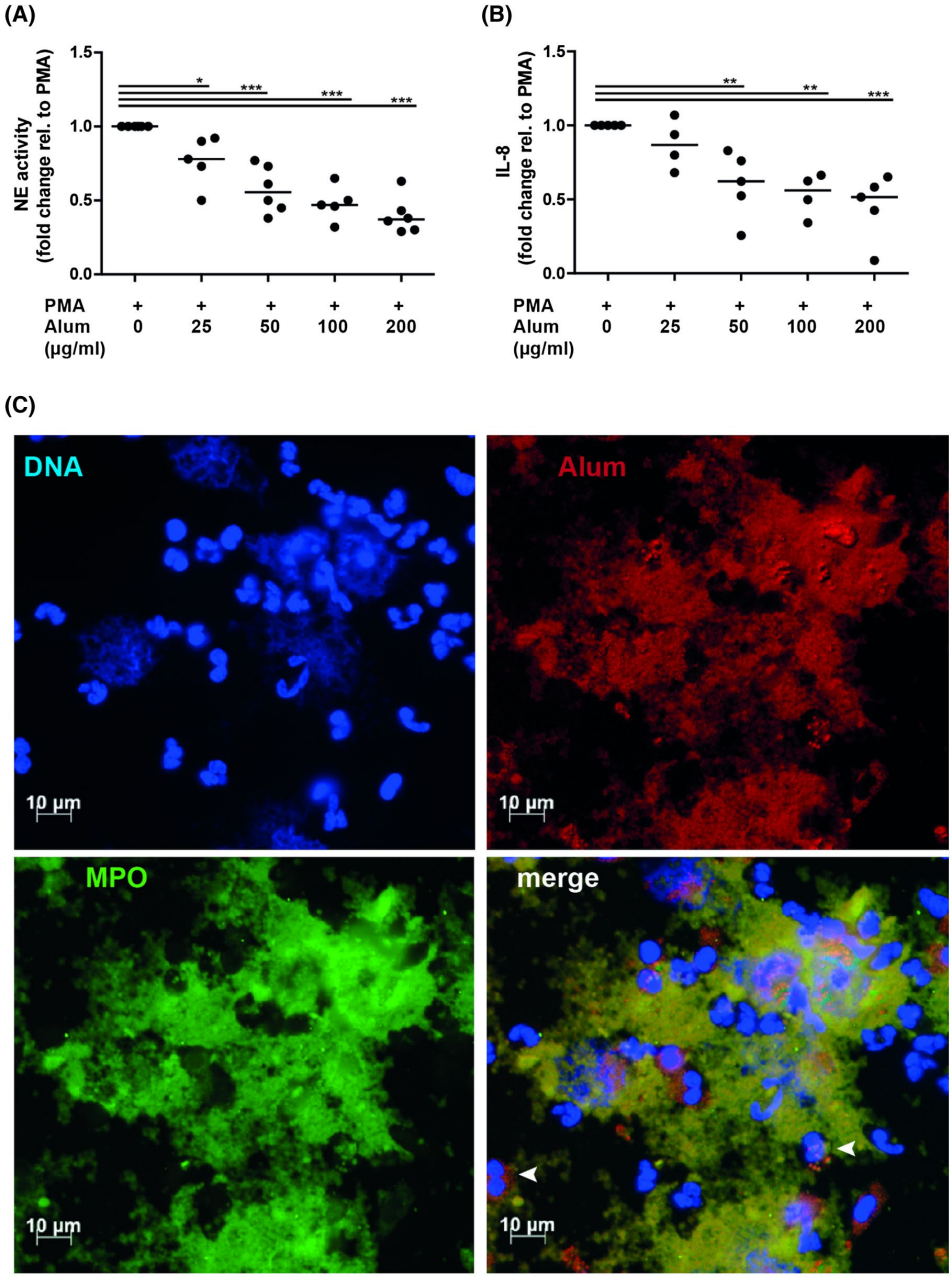
Interaction of alum with neutrophil‐derived material. Neutrophils were stimulated for 3 h with PMA alone or with PMA plus alum at the indicated concentrations and mildly treated with MNase. A, Elastase activity in the supernatants was quantified colorimetrically using N‐methoxysuccinyl‐Ala‐Ala‐Pro‐Val p‐nitroanilide as substrate (n = 5‐6). B, the presence of IL‐8 and in supernatants was determined by ELISA (n = 4‐5). *P < .05; **P < .01; ***P < .001 by One‐way ANOVA for repeated measurements followed by Dunnett's post tests. C, Fluorescence microscopy of neutrophils after 3 h of stimulation with 100 µg/mL lumogallion‐labeled alum illustrating the interaction of alum particles with NETs. Arrows indicate cells shortly after phagocytosis of alum particles, but still displaying lobulated nuclei. DNA, blue; MPO, green; alum, red; scale bar, 10 µm

Next, we analyzed the kinetics and extent of NET‐release with SYTOX orange, a cell membrane‐impermeant DNA‐dye. Alum triggered a concentration‐dependent NET‐release starting after 25 minutes of stimulation, similar to ionomycin (Figure 2B,C). Using extracellular NE‐activity in isolated NETs to quantify NET‐release, elevated levels of enzyme activity were observed with PMA, ionomycin and 25 or 50 µg/mL alum (Figure 2D). In contrast to DNA‐release, higher concentrations of alum resulted in lower NE‐activity, probably due to the potent ability to adsorb proteins, as the presence of alum in PMA‐stimulated neutrophils also dose‐dependently diminished NE‐activity (Figure 3A). Similarly, IL‐8 levels in these culture supernatants were also reduced (Figure 3B).

Monitoring the effect of alum on neutrophils by light microscopy, a gradual loss of cellular structures in most neutrophils suggested cell death. In line, we found that the number of viable CD66^+^CD16^+^ neutrophils decreased after 20 to 30 minutes (Figure S3) when tracking the alum‐induced cell loss by flow cytometry using counting beads, which accorded with the kinetics of DNA‐release (Figure 2B). In summary, alum‐induced NET‐formation and concomitant cytolysis.

### 3.4 NET‐induction depends on particle charge

**Figure 4:**
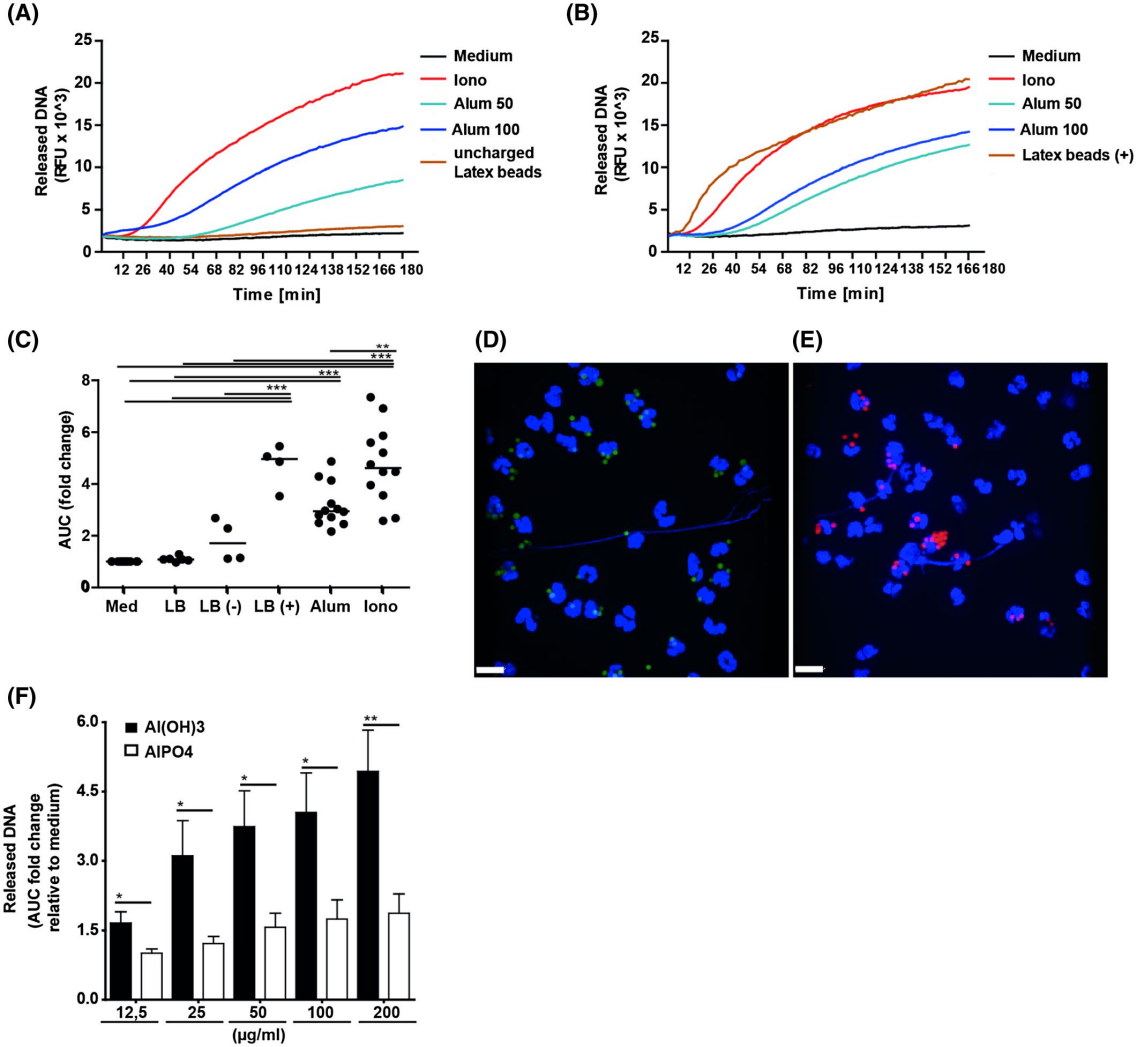
Positive particle charge is important for NET‐formation. Neutrophils were stimulated with ionomycin (5 µM), alum (50 and 100 µg/mL), or 100 µg/mL of (A) uncharged or (B) positively charged latex beads (Ø 3 µm). The kinetics of two representative experiments are shown. C, Cumulative data from four different donors. AUC, area under the curve; RFU, relative fluorescence units; horizontal bar, median. *P < .05; **P < .01; ***P < .001 by One‐way ANOVA for repeated measurements followed by Tukey's multiple comparison test. Fluorescence microscopy of neutrophils stimulated with (D) positively (green) or (E) negatively (red) charged latex beads. DNA (blue) was stained with DRAQ 5 (scale bar, 10 µm). F, DNA‐release induced by aluminium phosphate and aluminium hydroxide at equimolar Al^3+^ concentrations (n = 5). *P < .05; **P < .01; by paired t test

Scanning electron microscopy revealed nanoparticles and agglomerates with diameters of 1‐2 µm particle size in the alum preparation used for this study. A zeta potential of + 13.0 ± 3.5 mV (mean ± SD; n = 3) indicated positive surface charge (Figure S4). Accordingly, uncharged and positively charged latex particles of similar size were tested for their capacity to induce NETs. Rapid DNA release was observed in response to positively charged latex beads, whereas uncharged beads lacked this effect (Figure 4A‐C). NET‐formation was confirmed by fluorescence microscopy and expelled DNA was obtained in the presence of positively and to a lower extent also with negatively charged latex beads (Figure 4D,E). In contrast, uncharged beads were phagocytosed by neutrophils, but did not induce NET‐release (not shown). As latex beads differ from alum particles in shape and surface structure, we additionally tested NET‐induction by aluminium phosphate which also forms colloids and particles of similar diameter, but is negatively charged at neutral pH.^1^ At equimolar Al^3+^ concentrations, it induced detectable DNA‐release with similar kinetics, however 2.0‐2.5 times less than alum (Figure 4F). Thus, positive particle charge promoted NET‐induction.

### 3.5 Alum‐triggered NET‐formation involves production of mROS, citrullination of histones and release of nuclear and mitochondrial DNA

**Figure 5:**
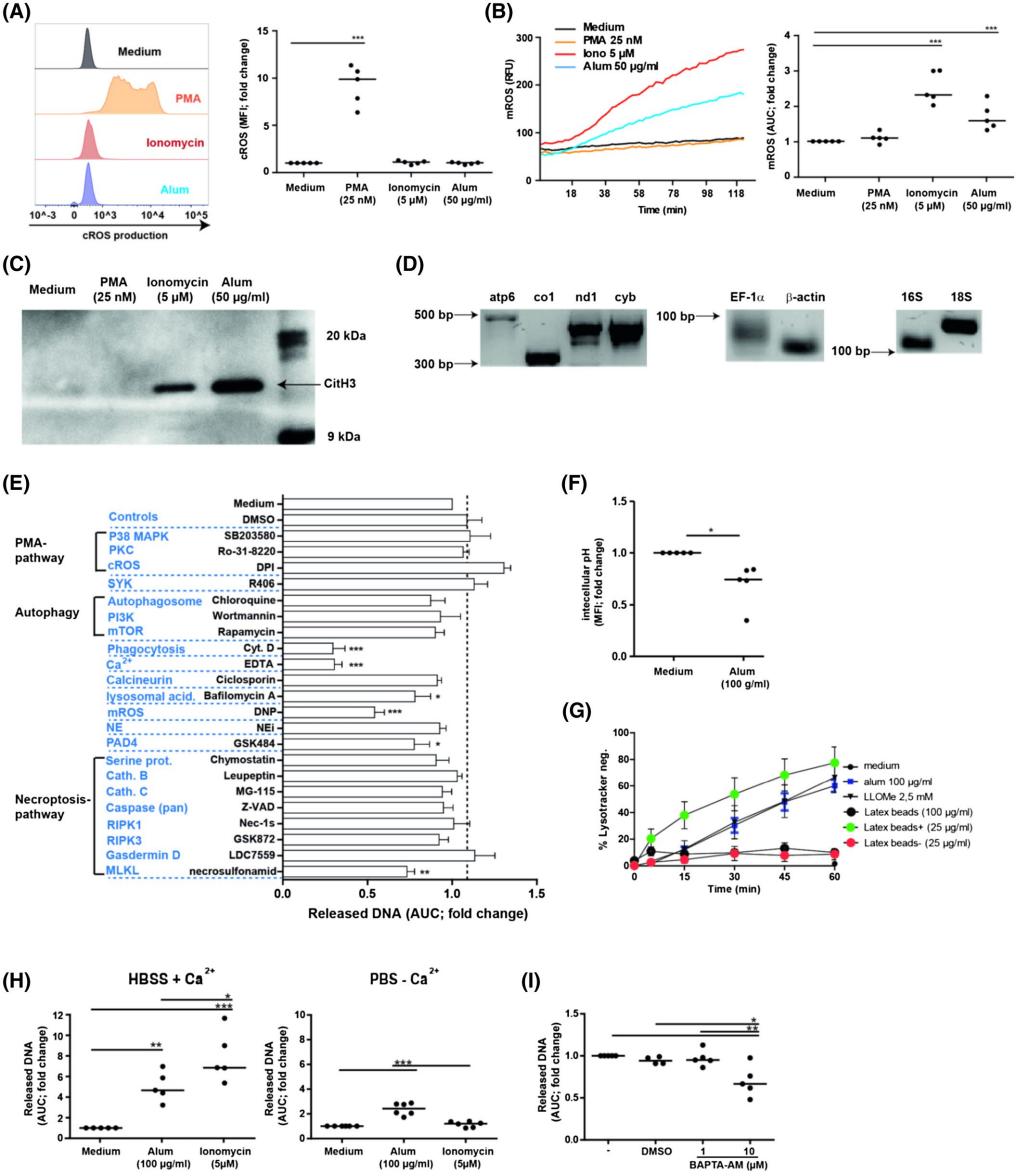
Pathways involved in alum‐triggered NET‐formation. A, Production of cROS by CM‐H_2_DCFDA‐loaded neutrophils 30 min after addition of the indicated stimuli as analyzed by flow cytometry. One representative experiment and cumulative data of five independent experiments using neutrophils from different donors are shown (n = 5). B, Production of mROS by neutrophils incubated with the indicated stimuli was assessed by addition of MitoSOX Red using plate reader assays (n = 5). C, Immunoblot analysis of citrullinated histone 3 (CitH3) in supernatants of MNase‐treated neutrophils after 3 h of stimulation with the indicated substances. BM; biotinylated protein marker. D, Isolated NET‐DNA was analyzed by PCR for the presence of mitochondrial (atp6, ATP synthase subunit 6; co1, cytochrome oxidase c subunit 1; nd1, NADH dehydrogenase subunit 1; cyb, cytochrome oxidase b; 16S, prokaryotic ribosomal subunit) and nuclear (EF‐1α, elongation factor 1 alpha; β‐actin and 18S, eukaryotic ribosomal subunit) genes. E, Alum‐induced NET‐formation in neutrophils which had been preincubated for 30 min with the indicated specific inhibitors or rapamycin (an inducer of mTOR and autophagy) (n = 5). F, The relative change of the intracellular pH in neutrophils preloaded with 3 µM BCECF, 5 min after stimulation with the indicated substances (n = 5). G, Neutrophils were loaded with 100 nM lysotracker and stimulated for different time points with alum, L‐leucyl‐L‐leucine methyl ester (LLOMe) a lysosomal destabilizing agent or latex beads as indicated (n = 5, mean ± SEM). H, Neutrophils were stimulated with alum or ionomycin for 3 h in the presence or absence of calcium and NET‐formation was measured by DNA‐release assay (n = 5). I, NET‐formation in neutrophils which had been preincubated for 30 min with a specific inhibitor for intracellular calcium (BAPTA‐AM) for 30 min, and stimulated with alum for 3 h (n = 5)

To elucidate the signaling pathway of alum‐induced NET‐release, the source of ROS production was analyzed. Significant cROS production in neutrophils preloaded with CM‐H_2_DCFDA was only observed after stimulation with PMA (Figure 5A), while production of mROS in neutrophils preloaded with MitoSOX Red was observed after stimulation with alum or ionomycin with very similar kinetics (Figure 5B). Hypercitrullination of histones, also assumed to be involved in NOX2‐independent NET‐induction^22^, ^31^ was detected by immunoblots in ionomycin‐ and alum‐induced, but not in PMA‐stimulated NETs (Figure 5C).

PCR with primers for mitochondrial and nuclear genes performed with alum‐induced NET‐DNA revealed the presence of both, mitochondrial and nuclear DNA (Figure 5D). Presence of the latter again indicated cell death, that is, NET‐formation in response to alum.^32^, ^33^

### 3.6 Pathways underlying alum‐triggered NET‐formation

To characterize the alum‐induced cellular mechanisms leading to NET‐formation, specific inhibitors of described NET‐inducing pathways were applied (Figure 5E).^34^, ^35^, ^36^, ^37^, ^38^ Inhibitors of the PMA‐induced signaling pathway, the receptor‐induced Syk‐dependent pathway or autophagy had no effect. Addition of EDTA reduced NET‐induction and inhibition of Ca^2+^‐dependent phagocytosis with cytochalasin D reduced NET‐release drastically. Phagocytosis was corroborated by the rapid increase of intracellular acidification found in alum‐stimulated neutrophils loaded with the pH‐sensitive reporter molecule BCECF‐AM^39^ (Figure 5F). The inhibitory effect of Bafilomycin A further indicated an important role of lysosomal acidification. Staining with LysoTracker (Figure 5G) demonstrated lysosomal rupture, similar to the effect of alum in macrophages.^40^ Positively charged latex beads led to even more pronounced lysosomal destabilization, in line with DNA‐release experiments (Figure 3C). As lysosomes store Ca^2+^ at mM levels, we hypothesized that upon rupture this Ca^2+^ supply would be sufficient to induce DNA‐release. In fact, DNA‐release was inducible by alum in Ca^2+^‐free PBS, whereas ionomycin, which requires extracellular Ca^2+^ was not effective (Figure 5H). Application of the intracellular Ca^2+^‐chelator BAPTA‐AM additionally decreased this DNA‐release in Ca^2+^‐free PBS significantly (Figure 5I). Moreover, inhibition of DNA‐release by Bafilomycin A, which leads to lower lysosomal Ca^2+^ concentrations by several orders of magnitude^41^ corroborated an essential role of lysosomal Ca^2+^ in alum‐induced NET‐formation.

Elevated intracellular Ca^2+^ levels are usually sensed by mitochondria which respond with increased respiration and the release of mROS as by‐products. Ca^2+^‐signaling via calcineurin in this process was excluded by the addition of ciclosporin A. Inhibition of mROS formation by the protonophore (2,4‐dinitrophenol; DNP) confirmed their importance for NET‐formation. Subsequent activation of PAD4 by mROS^42^ was suggested by the inhibitory effect of PAD4‐inhibitor GSK484 and the citrullinated histones observed in NETs as demonstrated in Figure 5C. Notably, specific inhibition of NE had no effect on DNA‐release (Figure 5E).

The release of NETs induced by PMA or urate crystals downstream of ROS production has been reported to involve the necroptosis pathway.^31^, ^43^ Interestingly, inhibitors of lysosomal proteases and caspases and of the farthest downstream molecules RIPK1, RIPK3, and MLKL described to trigger necroptosis, did not block DNA‐release with the exception of the MLKL‐blocker necrosulfonamide. In addition, a possible RIPK1‐ and RIPK3‐independent activation of MLKL by gasdermin D^44^ was excluded by inhibition experiments with LDC7559. Together, these results indicated that alum‐triggered NET‐formation depended on phagocytosis, Ca^2+^‐flux, lysosomal rupture, production of mROS, and PAD4 activity.

### 3.7 Metabolic changes during alum‐induced NET‐response

**Figure 6:**
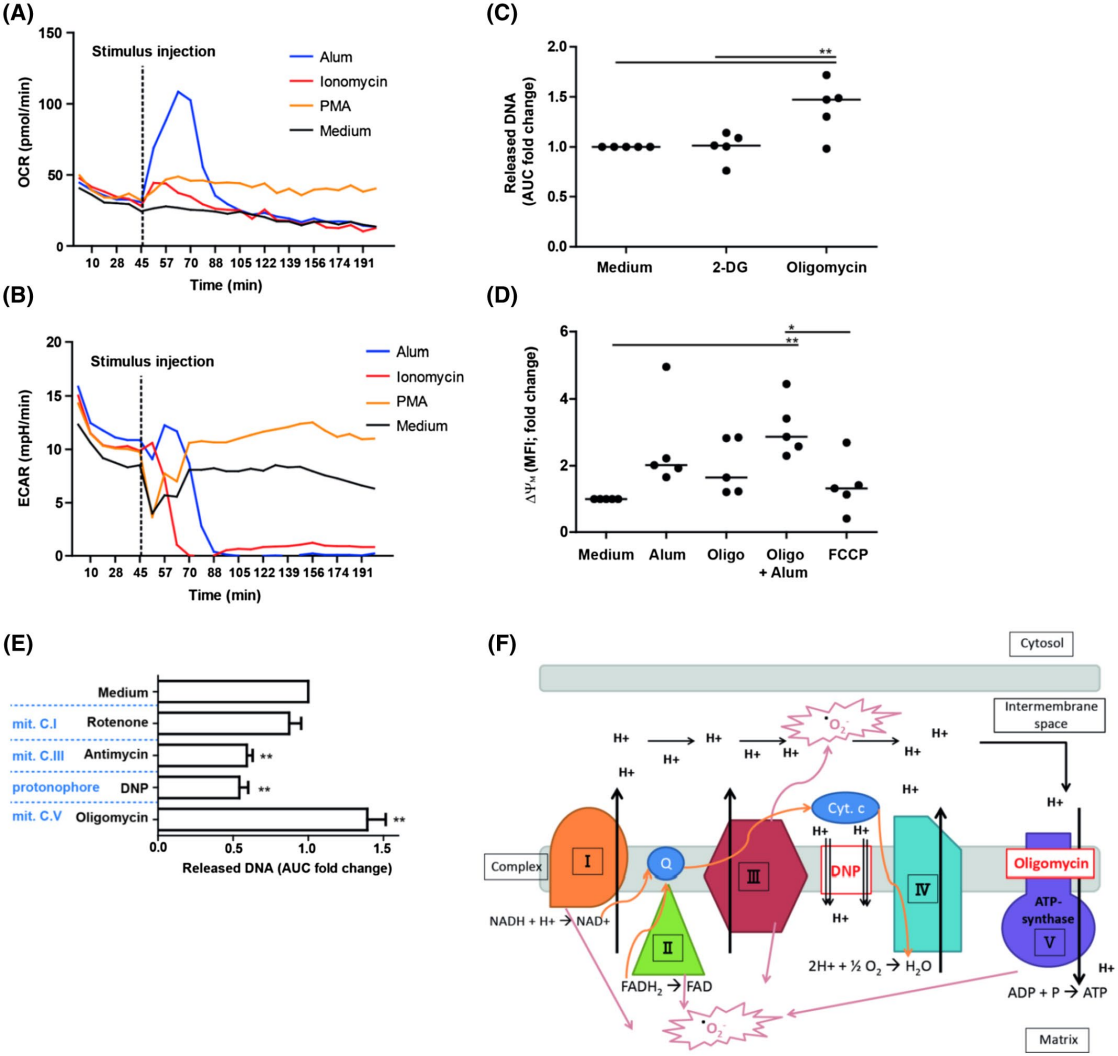
Metabolic changes in neutrophils during NET‐formation. Neutrophils were seeded in XF 24‐well microplates of an extracellular flux analyzer (Seahorse XFe24) and after a settling time of 45 min alum, ionomycin or PMA were injected. The kinetics of the extracellular oxygen consumption rate (OCR) (A) and the extracellular acidification rate (ECAR) (B) were recorded for an additional 155 min. The means of quadruplicates of each experiment were calculated and the means of five individual experiments with neutrophils from five different donors are shown. C, Alum‐induced DNA‐release of neutrophils (n = 5) in the presence of inhibitors for glycolysis (2‐DG, 2‐desoxy‐glucose; 2 mM) or oxidative phosphorylation (oligomycin; 10 µg/mL). D, Neutrophils were treated with oligomycin (Oligo) for 15 min before cells were incubated with 50 nM TMRE, an indicator of the mitochondrial membrane potential ΔΨ_m_, for 20 min. Then, cells were stimulated with alum (100 µg/mL) and within 5 min ΔΨ_m_ was assessed by flow cytometry (n = 5). The uncoupler of oxidative phosphorylation carbonyl cyanide‐*p*‐(tri‐fluromethoxy)phenyl‐hydrazone (FCCP), a potent protonophore, was used as negative control. E, Summarizes the effect of different mROS inhibitors on alum‐induced DNA release. Cumulative data of five independent experiments with neutrophils from different donors is shown. F, Schematic illustration of mitochondrial respiration and mROS production. During mitochondrial respiration mROS are produced within the electron transfer chain at complexes I, II, III, and V. This electron transfer is coupled to the translocation of protons across the inner mitochondrial membrane at complexes I, III, and IV which generates the mitochondrial membrane potential (ΔΨ_m_). Complex V normally utilizes this gradient for the phosphorylation of ADP to ATP, while protons flow back to the mitochondrial matrix. Oligomycin inhibits complex V leading to hyperpolarization of the membrane and to increased ΔΨ_m_. DNP uncouples oxidative phosphorylation from the electron transport chain. It acts as protonophore, that is, transports protons back to the mitochondrial matrix leading to the dissipation of the ΔΨ_m_

Neutrophils mainly depend on glycolysis for ATP‐production as their mitochondria are not fully functional.^45^, ^46^ Moreover, the production of mROS during NET‐formation indicated substantial mitochondrial activity (Figure 5B,E). The use of extracellular flux technology allowed us to simultaneously track the oxygen consumption rate (OCR; usually indicating respiration), and the extracellular acidification rate (ECAR; indicating glycolysis) during NET‐formation in real time. Alum, PMA, ionomycin, or medium were added to neutrophils and the metabolic changes during NET‐formation were recorded. Alum and ionomycin induced a transient increase in OCR within the first 30 minutes, with alum being the more potent stimulus (Figure 6A). A delayed increase with a stable plateau was obtained for PMA (Figure 6A). The early increases in OCR after addition of alum or ionomycin corresponded to the early production of mROS observed during NET‐release (Figure 5B), while the delayed OCR increase with PMA reflected the intracellular cROS production by NOX2.

In parallel, a transient increase of ECAR was obtained in alum‐ and ionomycin‐stimulated neutrophils suggesting glycolytic activity (Figure 6B). The striking drop of ECAR immediately after the addition of medium or PMA to neutrophil cultures most probably was caused by sample dilution and, therefore, the ECAR‐values for alum and ionomycin may actually be higher than observed. After about 10‐25 minutes, when DNA‐release sets in (Figure 2B; Figure S3), the extracellular flux of protons dramatically dropped below basal levels in alum‐ and ionomycin‐stimulated cultures reflecting the release of acidified cellular contents during cytolysis (Figure 6B).

### 3.8 The mitochondrial membrane potential plays an essential role in alum‐induced NET‐formation

We performed inhibition experiments to substantiate the results from extracellular flux analysis. Interestingly, the alum‐induced DNA release was not reduced by preincubation with 2‐desoxy‐glucose (2‐DG), an inhibitor of glycolysis, or with oligomycin (Figure 6C), an inhibitor of oxidative phosphorylation at complex V (F_o_F_1_‐ATPase) of the respiration chain (Figure S5A). Hence, there was no substantial need for replenishment of ATP by glycolysis or mitochondrial respiration for NET‐release. Unexpectedly, oligomycin even led to significantly increased DNA release. Pretreatment with oligomycin also induced significantly increased mROS production in response to stimulation with alum (Figure S5A,B). We, therefore, assume that the inhibition of oxidative phosphorylation at complex V also led to inhibition of the homeostatic retrograde proton transport executed by the ATP synthase. In turn, this would result in the accumulation of protons, that is, hyperpolarization of the inner mitochondrial membrane. In line, using the cell permeant fluorescent dye TMRE a significant increase of the mitochondrial membrane potential (ΔΨ_m_) was detected in oligomycin pretreated cells within 5 minutes of stimulation with alum (Figure 6D). As the dissipation of the ΔΨ_m_ by the protonophore DNP inhibited the DNA‐release (Figure 5E), we regard the increase of ΔΨ_m_ as a driving force for production of mROS leading to NET‐formation.

As neutrophils have impaired mitochondrial functions, we intended to identify the mitochondrial membrane complexes involved in alum‐induced mROS production and NET‐release. Inhibition of complex I with rotenone had no effect, whereas antimycin, an inhibitor of complex III, decreased DNA‐release to a similar extent as DNP (Figure 6E). Blocking of complex V by oligomycin increased the ΔΨ_m_ and hence amplified DNA‐release (Figure 6D).

## 4 DISCUSSION

Alum is the most frequently used adjuvant in human vaccines, but its mode of action is still not totally understood. In mice, NETs have been reported to be involved in the adjuvant effect of alum.^13^, ^16^ In humans neutrophils are five times more frequent in circulating white blood cells and could also play an important role for immune responses to alum‐adjuvant vaccines by releasing NETs as DAMPs to initiate innate responses. As a first step to reproduce the findings in mice in humans we used a novel human ex vivo skin model. Moreover, we identified the signaling processes which are involved in alum‐induced NET‐formation and added novel insights into the bioenergetic background of this programed cell death mechanism in neutrophils.

Similar to in vivo observations in mice,^13^ injection of alum particles into human skin biopsies led to infiltration of neutrophils from small vessels located in close vicinity of alum patches deeper in the dermis. Co‐staining of CitH3 and DNA indicated NET‐formation and suggests that in humans alum induces similar effects as in mice. In contrast, injection of PBS elicited the appearance of lower numbers of neutrophils closer to the epidermis, but without any NET‐formation. This example showed that neutrophils remaining in a 6 mm biopsy are sufficient to demonstrate their migration into tissue within 3 hours of incubation ex vivo. Similarly, human skin biopsies may be used to study early immune responses to sterile tissue damage or skin infections involving NET‐formation.

We also greatly expand the knowledge on cellular mechanisms involved in alum‐induced NET‐formation by in vitro experiments. Alum induced a NOX2‐independent NET‐release analogous to the Ca^2+^‐ionophore‐induced process, as it depended on Ca^2+^‐flux and the production of mROS, activation of PAD4 leading to citrullination of histones and decondensation of chromatin. Interestingly, other NET‐inducing particle‐sized materials like pristane or CaCO_3_/cholesterol crystals have been found to require NOX2‐dependent ROS as well as PAD4‐activation for NET‐induction in mouse models using Ncf1** (NOX2 mutant) and PAD4 ko mice.^47^, ^48^ Here we demonstrate that alum particles cause NOX2‐independent NET‐formation, as in contrast to mROS‐ cROS were not detectable and the NOX2 inhibitor DPI did not block alum‐triggered NET‐formation in human neutrophils. Similar to published data using PAD4 ko mice,^13^ experiments with Ncf1** mice should be performed in future studies to confirm our human in vitro data in vivo.

**Figure 7:**
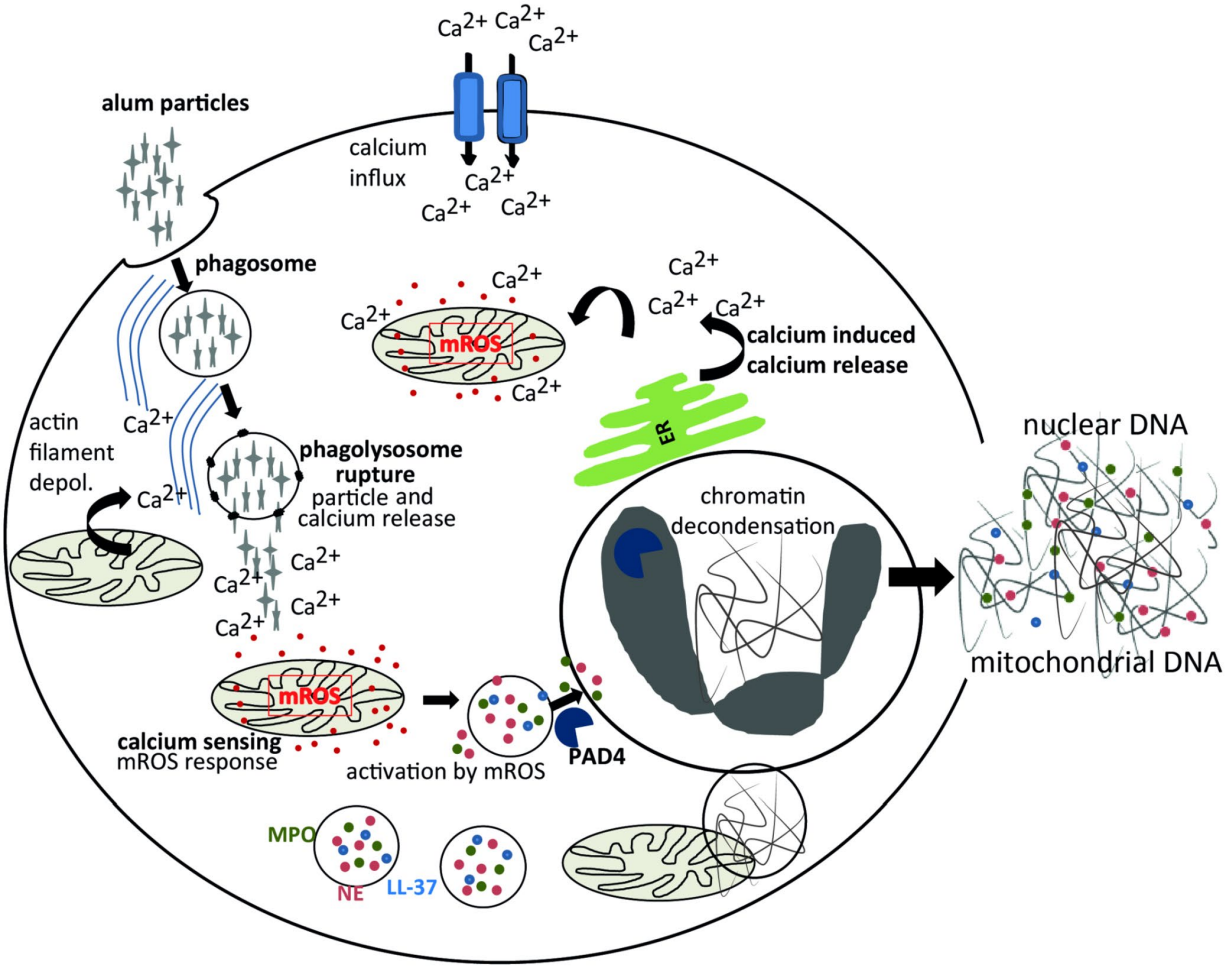
Proposed mechanism of alum‐induced NET‐formation. Neutrophils phagocytose alum particles. This actin‐dependent process requires Ca^2+^ for the depolymerization of actin filaments supplied by intracellular stores and secondary Ca^2+^ influx.^61^ During phagosome maturation alum destabilizes the phagolysosomal membrane resulting in the release of the lysosomal content including Ca^2+^ into the cytoplasm.^14^ The resulting elevated Ca^2+^ levels lead to oxidative mitochondrial stress and thereby to increased production of mROS.^51^ Besides, Ca^2+^ also activates the Ca^2+^‐dependent enzyme PAD4 leading to the decondensation of chromatin. Eventually, entropic chromatin swelling of the chromatin leads to rupture of the nuclear and plasma membrane and represents the driving force of the NET‐release ^53^

In contrast, to PMA or *Candida albicans*,^49^ and similar to the Ca^2+^‐ionophore A23187,^31^ alum did not require MPO/NE‐activity for chromatin decondensation. The cytosolic Ca^2+^ required for the phagocytosis of alum particles with actin‐dependent cellular transport activities could be provided by typical Ca^2+^ stores, such as the endoplasmatic reticulum. It is known that upon phagocytosis Ca^2+^ levels dramatically increase in lysosomes together with the drop in pH.^41^ Promoted by their positive charge alum particles led to lysosomal rupture and release of high amounts of Ca^2+^ into the cytosol. This Ca^2+^‐increase was sensed by mitochondria resulting in oxidative stress, that is, an overproduction of mROS. A direct relation of lysosomal leakage and NET‐formation has been discussed for nanoparticle‐induced NOX2‐dependent NET‐formation Munoz et al^33^, ^50^ Yet, our data suggest lysosomal Ca^2+^ as mediator for mitochondrial crosstalk in NET‐release. An analog mechanism has been described for programed cell death in murine and human macrophages after lysosomal rupture by destabilizing agents like LLOMe or alum.^14^, ^51^, ^52^ The final steps of the NET‐formation pathway described for phagocytosed crystals or nanoparticles, which also cause lysosomal damage, have been related to the induction of necroptosis involving the activation of the necroptosis‐complex consisting of RIPK3 and MLKL by RIPK1.^26^, ^43^ Interestingly, for alum‐induced NET‐release, only the inhibition of MLKL by necrosulfonamid caused marked reduction of DNA‐release, while inhibition of RIPK1 and RIPK3 had no effect. In addition, the alternative activation of MLKL by gasdermin D,^44^ but also release of NETs directly via gasdermin D could be excluded by its inhibition. Therefore, we propose that alum‐induced NET‐release does not depend on the expected necroptosis pathway, but it rather relies on an alternative, recently proposed mechanism.^53^ It implies that a PAD4‐dependent, entropic chromatin swelling drives the rupture of membranes and expulsion of DNA in neutrophils. Of course, we are aware of the fact that many chemical inhibitors can also bind to additional targets with lower specificity leading to off target effects. However, a normal genetic approach using knock down or knock out methods to elucidate the signaling pathway in primary human neutrophils is not possible. In Figure 7 we illustrate the mechanisms underlying alum‐induced NET‐release.

The sizes of naturally occurring NET‐inducing polar particles like urate‐, cholesterol‐, or calcium carbonate crystals range from 2‐15 µm.^24^, ^26^, ^28^ Although these microparticles have different shapes, ranging from globular to needle like structures, all have been reported to induce necroptosis and DNA‐release.^26^, ^54^ Apparently also particle charge plays a central role. In line, we found that positively and to a much lower extent also negatively charged microbeads‐induced DNA‐release (Figure 4A‐D). Uncharged latex microbeads, however, had no such effect, confirming a previous report.^50^ We conclude that the positive charge of alum strongly promotes its NET‐inducing capacity, especially by enforcing the permeabilization of the lysosomal membrane. Incorporating key structural elements of alum may lead to the development of new adjuvants for example, positively charged, biodegradable particles with increased safety profile.

Investigation of the energy metabolism during NET‐formation was the second focus of our study. Neutrophils depend mostly on glycolysis for ATP generation,^55^ which has been described as sole energy source for PMA‐triggered NET‐release.^56^ Extracellular flux analyses seemingly indicated induction of glycolysis during the first 5 or 15 minutes after addition of alum or ionomycin, respectively, corresponding well to the early DNA‐release. However, in contrast to PMA‐stimulated NET‐release^56^ and C5a‐induced NET‐induction,^57^ preincubation of neutrophils with the glucose analog 2‐DG did not inhibit DNA‐release induced by alum, indicating that newly generated ATP by glycolysis was not required. Along this line, it has been shown that human neutrophils consume ATP for phagocytosis without replenishing it to the original levels.^55^ Therefore, we interpret the extracellular acidification we observed (Figure 6B) as proton export across voltage‐gated proton channels in the plasma membrane, triggered by the rapid cytoplasmatic acidification during early phagocytosis (Figure 5H).^39^

The involvement of mROS in alum‐induced NET‐formation and our extracellular flux experiments indicated activity of the respiratory chain. Unexpectedly, addition of oligomycin led to increased DNA‐release, again supporting the notion that generation of ATP by oxidative phosphorylation was also not required for NET‐release. Instead, by inhibiting the retrograde transport of H^+^ at mitochondrial complex V (Figure 7E), oligomycin increased the ΔΨ_m_ within 5 minutes of stimulation with alum, thus anticipating the production of mROS (Figure S6A,B). In analogy, studies with myocytes have revealed that increases of ΔΨ_m_ usually precede excessive mROS production.^58^ In our experiments, activity of complex III was mainly responsible for the increase of ΔΨ_m_, which was in line with the reported baseline maintenance of ΔΨ_m_ in neutrophils.^59^ Interestingly, it differed from NET‐induction by PMA^56^, ^60^ or C5a or LPS,^57^ which has been described to be dependent on complex I‐controlled, glycolytic ATP production. Thus, our study reveals a not yet recognized crucial role of the ΔΨ_m_ in neutrophils for induction of mROS and NET‐release without need for additional energy supply by glycolysis, supporting entropic chromatin swelling as driving force.

In summary, to the best of our knowledge we are first to demonstrate that injection of alum leads to neutrophil infiltration and NET‐formation in human tissue. This finding is similar to those described in mice and, therefore, encouraging to assume that in human NETs may contribute to the adjuvant effect of alum. Furthermore, we show that the essential requirements for alum‐induced NET‐formation are positive particle charge, lysosome‐mediated activation of mitochondria leading to hyperpolarization of the mitochondrial membrane providing mROS. As the released NET‐DNA binds to alum, it will be of great interest to investigate uptake and effects of this complex in APC, for example, intracellular DNA‐receptor activation and modulation of innate responses.

## CONFLICT OF INTEREST

The authors declare that they have no conflict of interest.

## AUTHOR CONTRIBUTIONS

M. Reithofer, B. Jahn‐Schmid, B. Bohle, K. Schmetterer, and G. Stary designed research; M. Reithofer, D. Pollak, C. Kitzmüller, and J. Strobl established methods, M. Reithofer, J. Karacs, K. Seif, M. Kamalov, G. Greiner, and J. Strobl performed research; M. Reithofer, B. Jahn‐Schmid., and CFW Becker analyzed data; M. Reithofer, B. Bohle, and B. Jahn‐Schmid wrote the paper.

## Supporting information

Fig S1‐S6

Supplementary Material
