# Supplementary figures and images for: Alum triggers infiltration of human neutrophils ex vivo and causes lysosomal destabilization and mitochondrial membrane potential‐dependent NET‐formation
Source: FASEB J. 2020 Aug 29;34(10):14024–41. doi: 10.1096/fj.202001413R (PMC7589265; doi:10.1096/fj.202001413R)

**Supplementary Figures (S1-S6)**


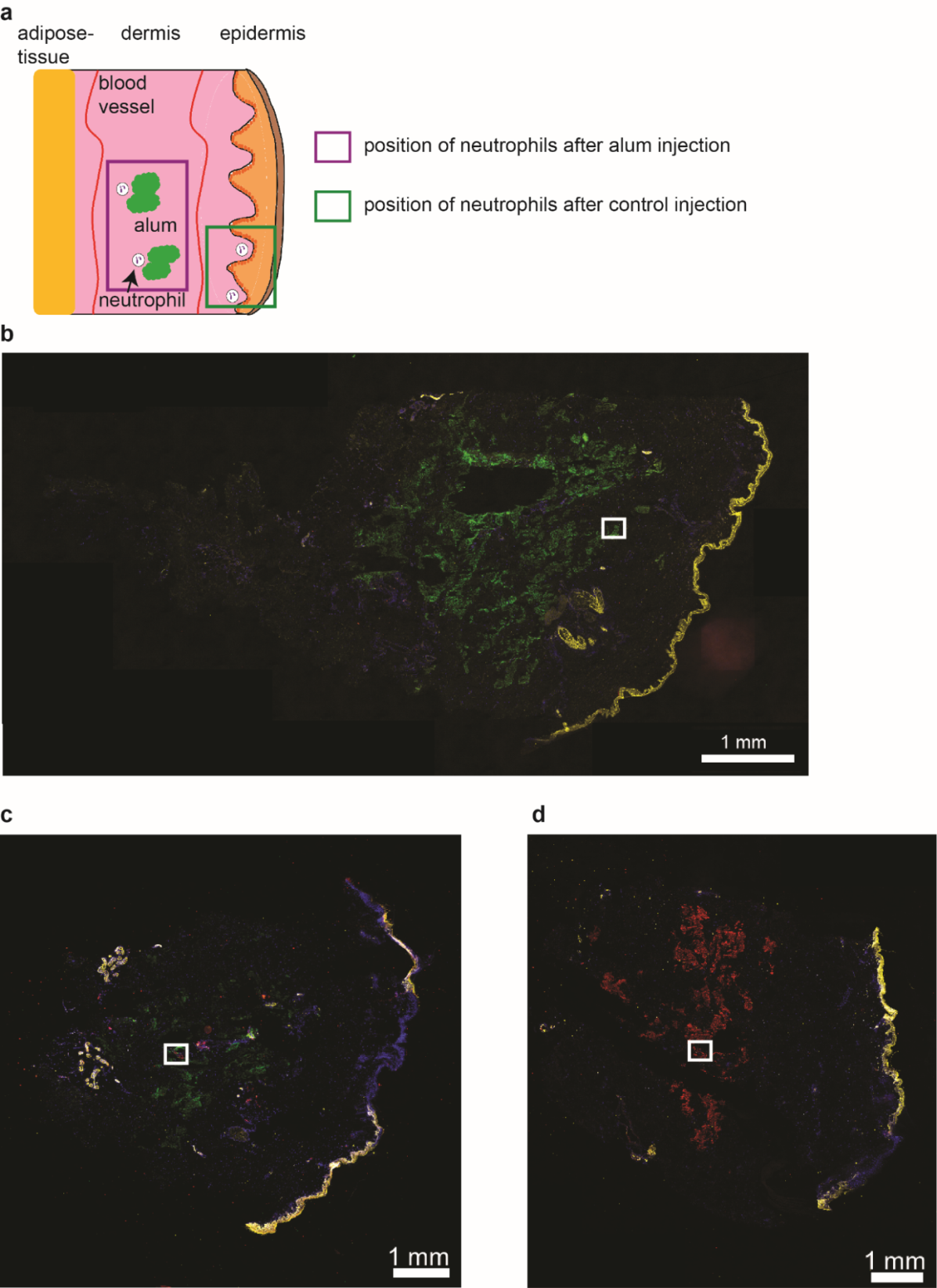


**Fig. S1**


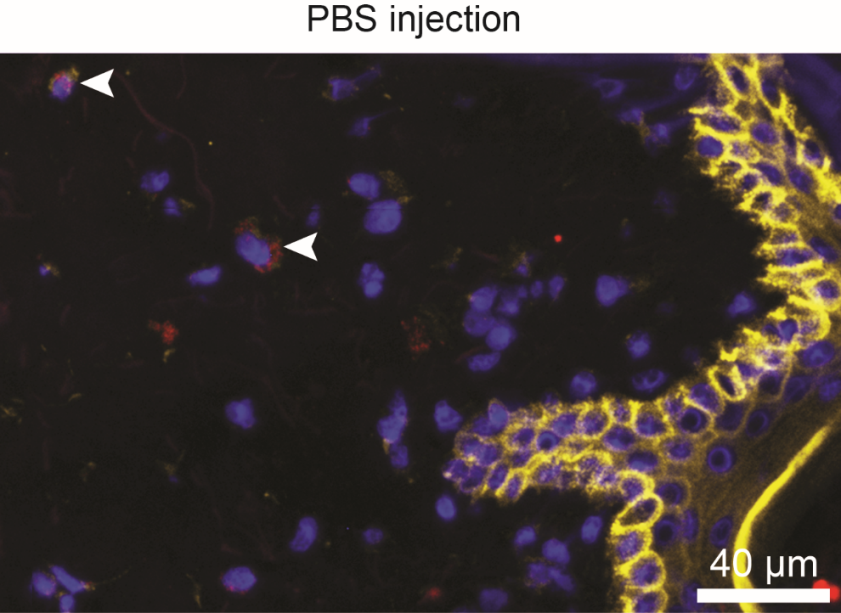


**Fig. S2**


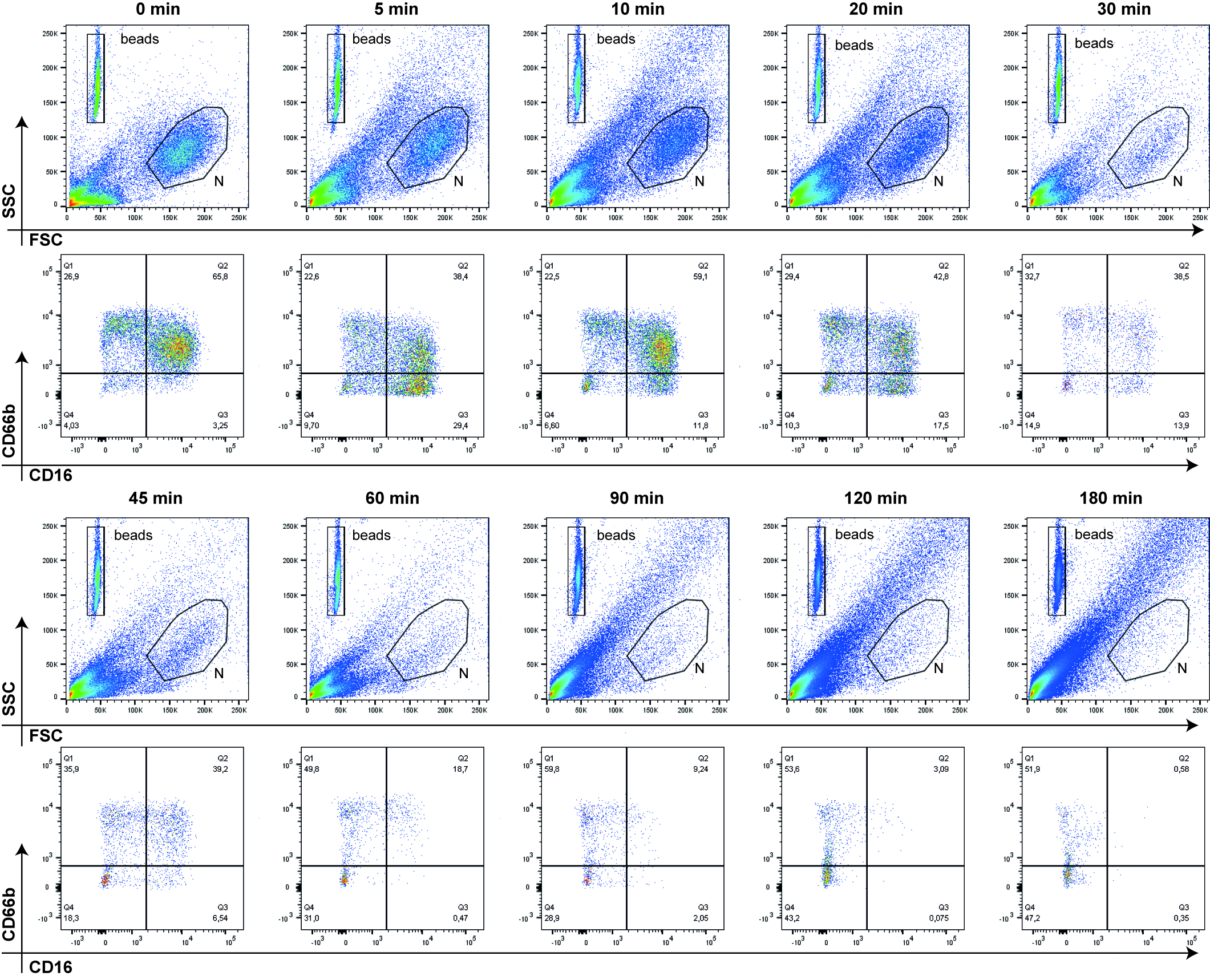


**Fig. S3**


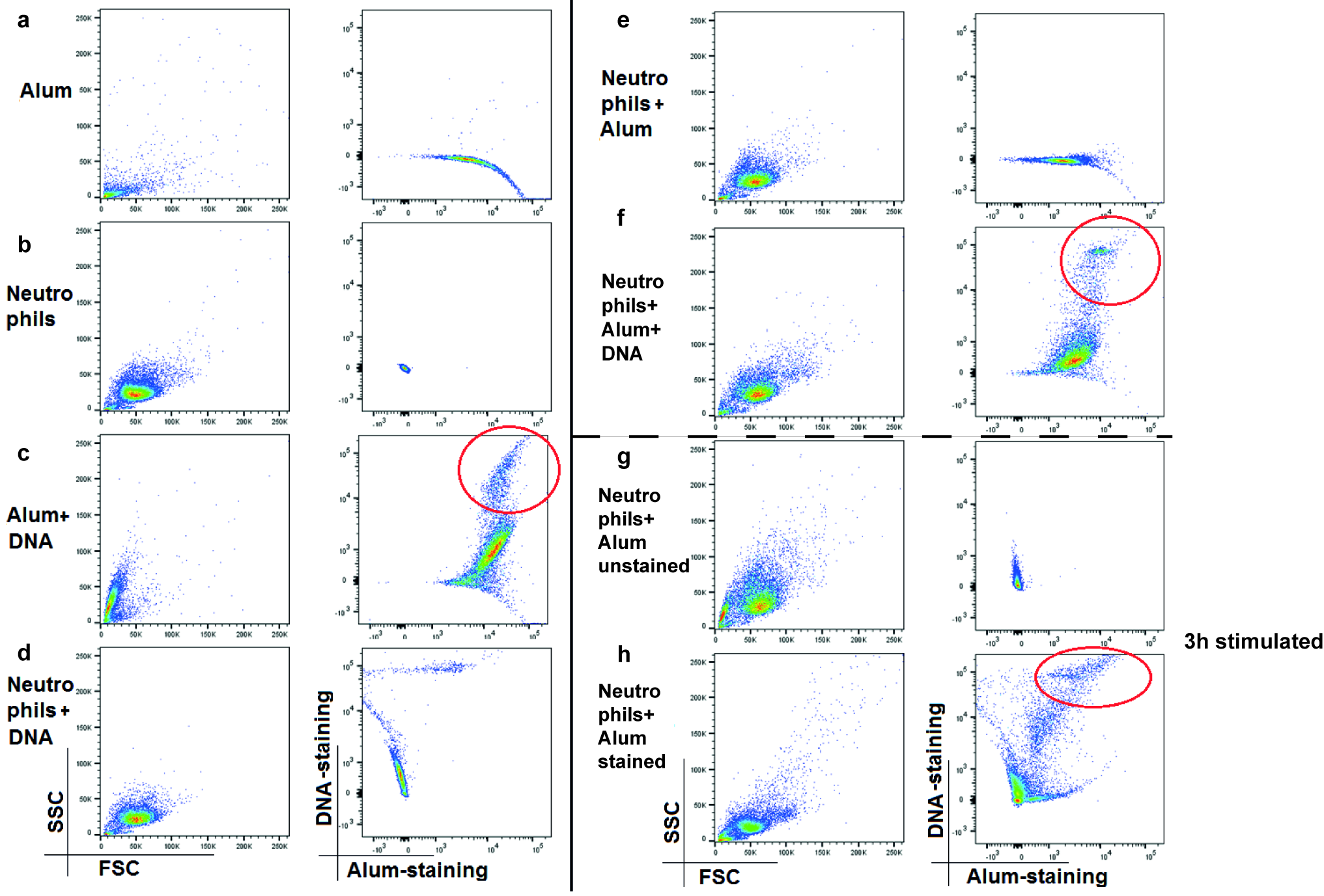


**Fig. S4**


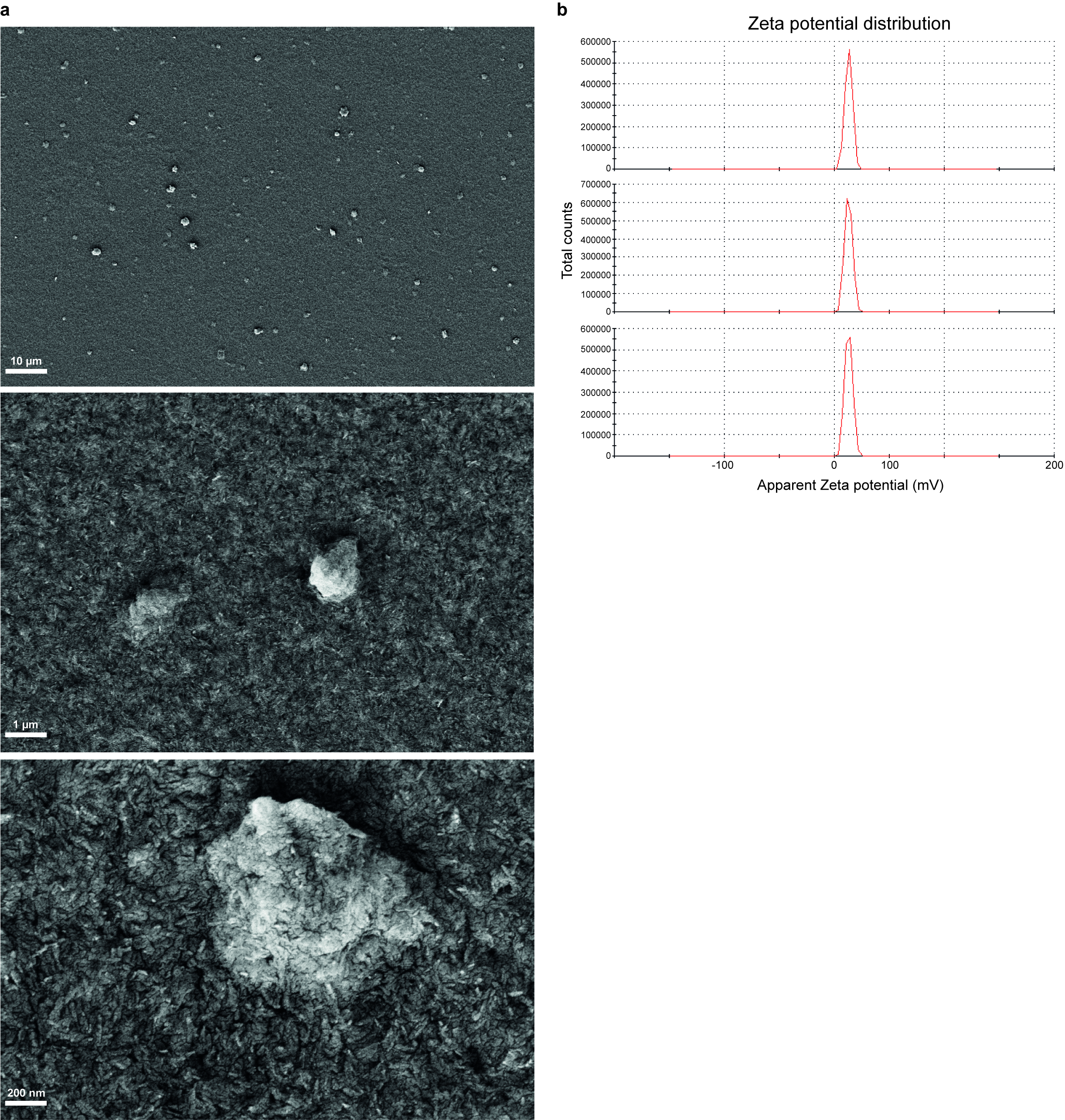


**Fig. S5**


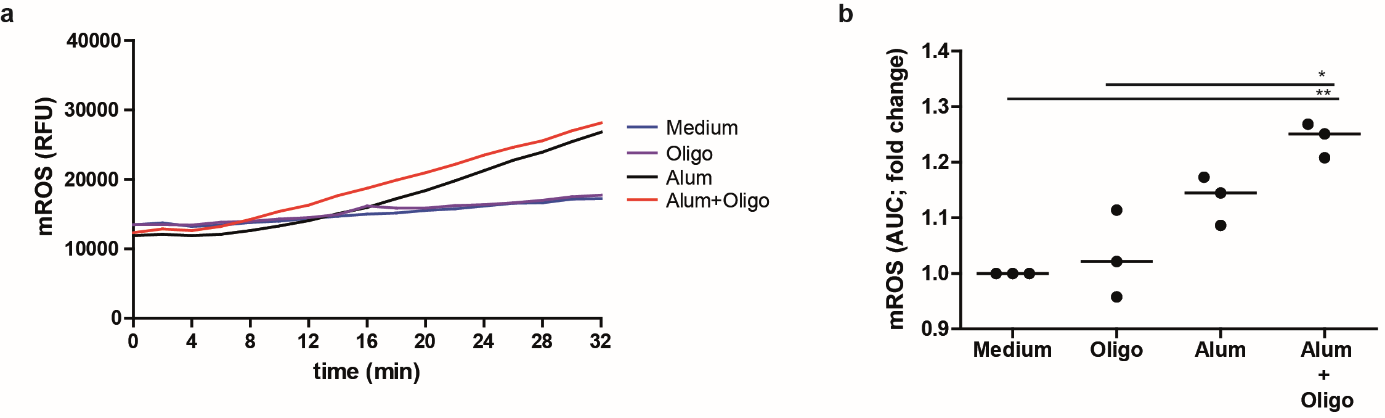


**Fig. S6**

Supplement: Supplementary file 1 — Fig S1‐S6 [file FSB2-34-14024-s001.docx]
