# Supplementary material for: Alum triggers infiltration of human neutrophils ex vivo and causes lysosomal destabilization and mitochondrial membrane potential‐dependent NET‐formation
Source: FASEB J. 2020 Aug 29;34(10):14024–41. doi: 10.1096/fj.202001413R (PMC7589265; doi:10.1096/fj.202001413R)
Supplement: Supplementary file 2 — Supplementary Material [file FSB2-34-14024-s002.docx]

**Supplementary Text**

**Aluminium hydroxide triggers infiltration of human neutrophils and causes mitochondrial membrane potential-dependent NETosis**

Manuel Reithofer^1^, Jasmine Karacs^1^, Johanna Strobl^2^, Claudia Kitzmüller^1^, Dominika Polak^1^, Katharina Seif^3^, Meder Kamalov^4^, Christian F.W. Becker^4^, Georg Greiner^5^, Klaus Schmetterer^5^, Georg Stary^2,6,7^, Barbara Bohle^1^, and Beatrice Jahn-Schmid^1^

^1^Institute of Pathophysiology and Allergy Research, Center for Pathophysiology, Infectiology and Immunology, Medical University of Vienna, Vienna, Austria

^2^Department of Dermatology, Medical University of Vienna, Vienna, Austria

^3^Department of Surgery, Medical University of Vienna, Vienna, Austria

^4^Institute of Biological Chemistry, Department of Chemistry, University of Vienna, Vienna, Austria

^5^Department of Laboratory Medicine, Medical University of Vienna, Vienna, Austria

^6^Ludwig Boltzmann Institute for Rare and Undiagnosed Diseases, Vienna, Austria

^7^CeMM Research Center for Molecular Medicine, Vienna, Austria

**Corresponding author:** Beatrice Jahn-Schmid

**E-mail:** [beatrice.jahn-schmid@meduniwien.ac.at](mailto:beatrice.jahn-schmid@meduniwien.ac.at)

**Supplementary information**

Supplementary figure legends: S1-S6

**Figure S1. *Ex vivo* injection of alum into human skin leads to attraction of neutrophils and extracellular trap formation.** (**a**) Schematic overview of a skin section with a frame showing the position of infiltrated neutrophils after injection of alum or PBS, respectively. (**b**)-(**d**) Representative images of IF staining of skin sections after injection of alum. White squares indicate the respective zoomed-in areas shown in Fig. 1 a, b and c, respectively. (**b**) Staining for DNA (blue), NE (yellow), CD16 (red) and alum (green). (**c**) Staining for DNA (blue), CitH3 (yellow), LL-37 (red) and alum (green). (**d**) Staining of DNA (blue), CitH3 (yellow) and alum (red). Scale bar 1 mm. The strong staining of the epidermis and glands are auto-fluorescence.

**Figure S2. Neutrophils present after *ex vivo* injection of PBS are located close to the border between epidermis (stratum basale) and dermis.** Representative image showing neutrophils (white arrows) stained with DNA (blue), NE (yellow) and CD16 (red) in control skin (n=3). Scale bar 40 µm. The strong staining of the epidermis is due to autofluorescence.

**Figure S3.** **Alum induces cell death of neutrophils**. Primed neutrophils were stimulated with alum (100 µg/ml) for the indicated time periods. To determine the loss of viable cells with time, cells were harvested, stained and analyzed by flow cytometry together with counting beads. 10,000 counting beads were acquired for each time point. FSC/SSC plots and gating of CD16^+^CD66b^+^ neutrophils (N) and counting beads are shown.

**Figure S4. Interaction of NETs with alum.** Alum/DNA complexes can be detected by flow cytometry and are indicated by red circles (**a,c,h**). Diverse controls: (**a**) fluorescence of lumo­gallion-labelled alum particles or (**b**) unstained neutrophils; DNA purified from neutrophil lysates stained with SYTOX green and mixed with (**c**) stained alum particles, or (**e**) with neutrophils or both; (**f**) stained alum particles mixed with neutrophils immediately analyzed. (**h**) To demonstrate alum/NET-DNA complexes, neutrophils were stimulated with lumo­gallion-labelled alum for 3 h, and DNA was stained by SYTOX green immediately before analysis. (**g**) Unstained negative control. One out of two experiments with similar results is shown.

**Figure S5.** **Particle sizes and zeta potential of alum**. (**a**) Scanning electron micrographs of alum (Alu-Gel-S) show conglomerates of nanoparticles and microparticles (magni­fications: 852x, 10.000x, 30.744x; scale bars: 10µm, 1µm, 200nm). (**b**) The apparent zeta potential of 0.01 mg/ml alum in water was measured three times.

**Fig. S6. Changes in mROS production by oligomycin.** Neutrophils were incubated with the indicated stimuli in the presence of the mROS-indicator MitoSOX™ Red and production of mROS was assessed by plate reader assays. (**a**) One representative experiment. (**b**) Cumulative data of 3 independent experiments using neutrophils from different donors.
